# BODYFAT: a new calculator to determine the risk of being overweight validated in Spanish children between 11 and 17 years of age

**DOI:** 10.1007/s00431-024-05596-2

**Published:** 2024-06-19

**Authors:** María Victoria Martín-Miguel, María Victoria Delgado-Martín, Carolina Barreiro-Arceiz, Ana Goicoechea-Castaño, Sara Rodríguez-Pastoriza, Clara González-Formoso, Martín Fernández-Pérez, Clara García-Cendón, Javier Roca, Ana Clavería

**Affiliations:** 1 Vigo Family and Community Medicine and Nursing Teaching Unit, Health Area of Vigo, SERGAS, Vigo, Spain; 2 Moaña Health Center, SERGAS, Vigo Area, Vigo, Spain; 3 Baiona Health Center, SERGAS, Vigo Area, Vigo, Spain; 4 Sárdoma Health Center, SERGAS, Vigo Area, Vigo, Spain; 5 I-Saúde Group, South Galicia Health Research Institute (Instituto de Investigación Sanitaria Galicia Sur), SERGAS-UVIGO, Vigo, Spain; 6 Ponteareas Health Center, SERGAS, Vigo Area, Vigo, Spain; 7 Val Miñor Health Center, SERGAS, Vigo Area, Vigo, Spain; 8 Galician Research and Mathematical Technology Center (Centro de Investigación e Tecnoloxía Matemática de Galicia/CITMAga) & Department of Statistics and Operations Research, University of Vigo, Vigo, Spain; 9 Research Netwpork in Chronicity, Primary Care and Health Promotion (Red de Investigación en Cronicidad, Atención Primaria y Promoción de la Salud/RICAPPS), Vigo, Spain

**Keywords:** Body composition, Anthropometry, Body fat, Body mass index, Children and adolescents, Overweight, Obesity

## Abstract

The assessment of body fat of children in primary care requires consideration of the dynamic changes in height, weight, lean mass, and fat mass during childhood growth. To achieve this, we aim to develop a predictive equation based on anthropometric values, with optimal diagnostic utility. This is a cross-sectional observational study, involving schoolgoers aged 11–17 years in the Vigo metropolitan area. Out of 10,747 individuals, 577 were randomly recruited. Variables: age, sex, ethnicity/country of origin, weight, height, 8 skinfolds, 3 diameters, 7 perimeters, and 85% percentile of body fat mass as the gold standard. Generalized additive regression was selected by cross-validation and compared using receiver operating characteristic curves (ROC curves). Sensitivity, specificity, positive and negative predictive values, true positive and true negative values, false positive and false negative values, accuracy, and positive and negative likelihood ratios were calculated. Two models were identified. The optimal model includes sex, weight, height, leg perimeter, and arm perimeter, with sensitivity of 0.93 (0.83–1.00), specificity of 0.91 (0.83–0.96), accuracy of 0.91 (0.84–0.96), and area under the curve (AUC) of 0.957 (0.928–0.986). The second model includes sex, age, and body mass index, with sensitivity of 0.93 (0.81–1.00), specificity of 0.90 (0.80–0.97), accuracy of 0.90 (0.82–0.96), and an AUC of 0.944 (0.903–0.984).

*Conclusion*: Two predictive models, with the 85th percentile of fat mass as the gold standard, built with basic anthropometric measures, show very high diagnostic utility parameters. Their calculation is facilitated by a complementary online calculator.

**Table:** 

**What is Known:** *• In routine clinical practice, mainly in primary care, BMI is used to determine overweight and obesity. This index has its weaknesses in the assessment of children.*	
**What is New:** *• We provide a calculator whose validated algorithm, through the determination of fat mass by impedanciometry, makes it possible to determine the risk of overweight and obesity in the community setting, through anthropometric measurements, providing a new practical, accessible and reliable model that improves the classification of overweight and obesity in children with respect to that obtained by determining BMI.*

**Supplementary Information:**

The online version contains supplementary material available at 10.1007/s00431-024-05596-2.

## Introduction

The surge in obesity prevalence over the past 25 to 30 years has been so pronounced that the World Health Organization (WHO) has deemed it “the pandemic of the twenty-first century.” Presently, over one billion individuals worldwide are obese, including 340 million adolescents and 39 million children [1]. This surge is not confined to high-income countries but is impacting low- and middle-income nations as well [2]. Projections suggest that the number of overweight breastfeeding infants and young children may rise to 70 million by 2025 [1].

The COVID-19 pandemic has exacerbated childhood obesity due to increased screen exposure, reduced physical activity, and shifts in dietary habits towards cost-effective, time-saving ultra-processed foods [3], contributing to a phenomenon termed “covidbesity” [4].

The term “obesity” refers to an excess of fat. Adiposity, commonly assessed using body mass index (BMI), stands as the predominant parameter in clinical practice for evaluating overweight and obesity in childhood and adolescence due to its easy applicability, cost-effectiveness, and non-invasiveness [5]. However, BMI, while widely used, does not provide a direct measure of body fat. Consequently, it may overestimate adiposity in children with increased muscle mass and, conversely, underestimate it in those with reduced muscle mass [6]. Additionally, the relationship between BMI and fat mass exhibits variations based on sex and age. Unlike the linear increase in BMI with age, fat mass tends to stabilize or even decrease between the ages of 8 and 12 [7]. A meta-analysis of studies involving children and adolescents revealed low sensitivity in detecting obesity when relying solely on BMI cut-off values. Notably, approximately one in four children with excess body fat remained unidentified as obese when assessed using BMI alone [8].

Various models, spanning from the atomic level to tissue, exist for determining body composition [6]. Generally, the most accurate methods are intricate, lack portability, demand an extended duration, and, in certain cases, involve exposure to radiation, incurring high costs [9–12]. Consequently, their application in clinical practice is limited, making them unsuitable for the broad follow-up or screening of large population groups, relegating them primarily to research.

The dynamic changes in height, weight, lean mass, and fat mass during children’s growth necessitate the adaptation of body fat assessment methods. DXA is considered gold standard for the determination of body composition. However, bioimpedance emerges as an alternative method due to its lack of radiation and its accessibility for outpatient evaluation [13]. Despite its utility, particularly in nutrition clinics due to its simplicity and strong correlation with cardiovascular risk factors, it is crucial to note that bioimpedance is currently infrequently employed in clinical practice within primary care settings.

The assessment of nutritional status frequently employs measurements of perimeters and skinfolds, which are integral to prediction equations aimed at estimating various body compartments. However, limitations within the anthropometric method, such as reduced accuracy in detecting short-term alterations and decreased precision in individuals classified as obese, have been acknowledged [14].

Compelling evidence demonstrates a robust correlation between individual measurements of skinfold thickness and fat mass as quantified by dual-energy X-ray absorptiometry (DXA) in child populations [15]. Anthropometric equations that include tricipital and subscapular skinfolds emerge as the most accurate [16].

Given the accessibility, ease of implementation, and safety of anthropometric measurements within primary care settings [17], this study endeavors to formulate a predictive equation based on anthropometric values. The objective is to assess body composition in a pediatric population, enabling optimal classification of associated overweight risk with optimal accuracy, sensitivity, and specificity. Additionally, an open-use calculator will be developed to enhance its practical application.

## Materials and methods

### Study design

This cross-sectional observational study aimed to assess anthropometric measurements among the schoolgoing population in Vigo. The study was conducted during May–June 2009.

### Participants

The study population included both male and female schoolgoers aged 11 through 17 years in the Vigo metropolitan area. The total schoolgoing population in Vigo was 10,747, with 60% attending state-subsidized schools and 40% public schools. The breakdown by school year was 2741 in the first year of Compulsory Secondary Education, 2789 in the second year, 2735 in the third year, and 2482 in the fourth year.

### Sample size

The sample size calculation was based on an estimated overweight prevalence of 17%, a 95% confidence interval, the ability to detect a difference of 3%, and a total schoolgoing population of 10,747 students. A total of 577 participants were to be recruited, as determined by a sample size calculator [18].

Cluster randomization was performed with schools as the sampling units, ensuring representation from each school year. In the sample, efforts were made to have each course represent 25% of the total.

The sampling procedure involved randomly selecting the initial school from a compiled list of both public and state-subsidized schools. Afterwards, consecutive schools were chosen until the predetermined sample size was achieved. In instances where a school declined participation, the subsequent school listed was then selected to ensure the continuation of the sampling process.

### Variables

The study encompassed various variables for comprehensive assessment: age (years), sex (male and female), nationality/country of origin, and height (cm); tricipital, bicipital, subscapular, suprailiac, abdominal, pectoral, thigh, and leg skinfolds (mm); radial bistyloid, humeral biepicondyle, femoral biepicondyle bone diameters (cm); waist, hip, contracted arm, relaxed arm, head, wrist, and leg girths (cm); and impedance measurement. Protocols detailing the measurement procedures are available in Appendix 1, and the instruments used are listed in Appendix 2.

The classification of overweight and obesity was determined based on the bioimpedance measurement criteria of Mueller et al., using the 85th percentile as the cut-off point for each age. This study refers to the 5th to 95th percentiles of body fat percentage, derived from bioelectrical impedance, of a cohort of 678 children of different races (black and non-black) who were followed for 4 years [19, 20].

### Data collection and analysis

Before initiating fieldwork, researchers underwent training to standardize the procedure for obtaining anthropometric measurements. Selected schools were briefed about the study, and information leaflets and informed-consent forms were distributed to pupils. The study criteria were explained to school authorities, teachers, and parents/guardians.

Anthropometric data were collected by a research team during scheduled visits to schools. Measures were directly entered into an EXCEL spreadsheet, with each pupil assigned a unique code for anonymity. Pupils without signed consent forms or those with illnesses affecting anthropometric values were excluded. Information about the measurements was provided to families upon request.

A comprehensive statistical analysis was conducted, involving data cleaning, debugging, and the removal of implausible values. For qualitative variables, absolute frequencies and percentages were presented, while for quantitative variables, normality was assessed, and mean and standard deviation (SD) or median and 25th and 75th percentiles were reported as appropriate.

**Fig. 1:**
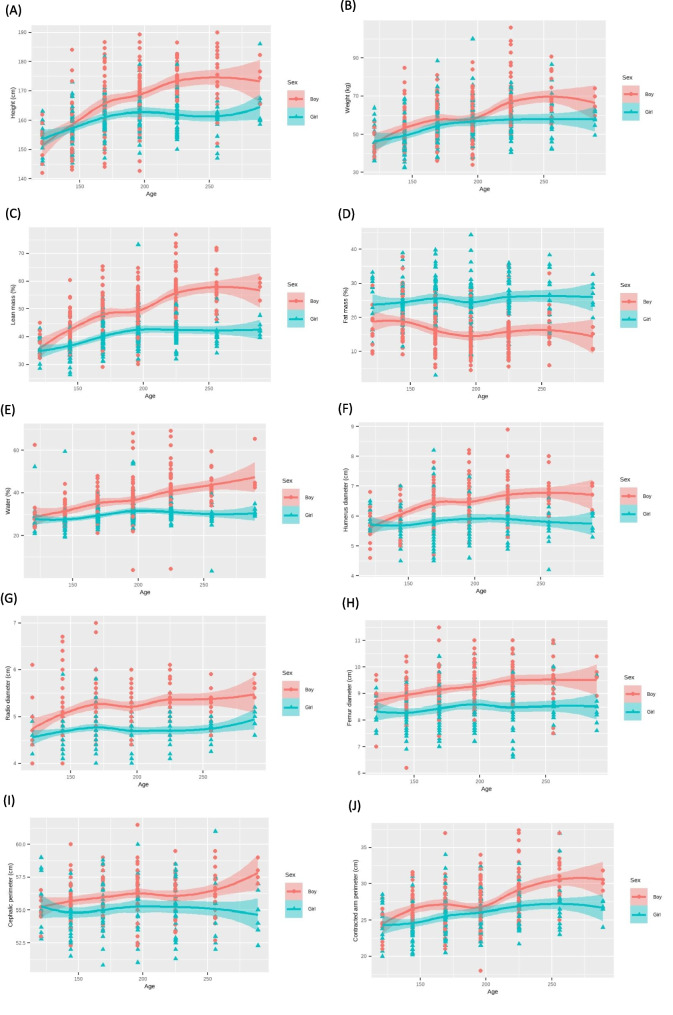

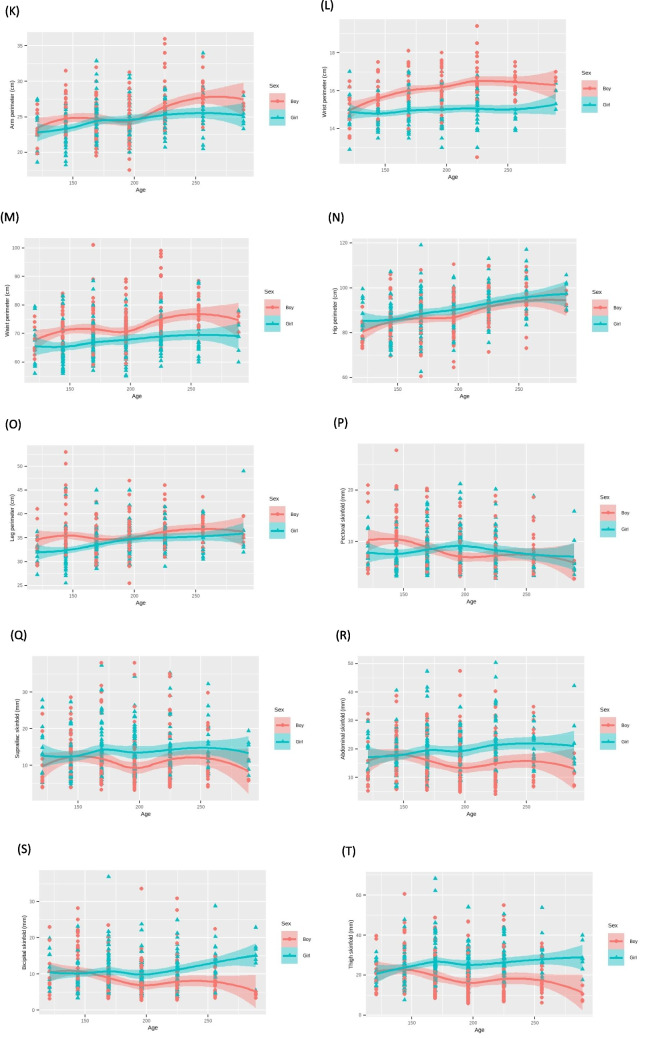

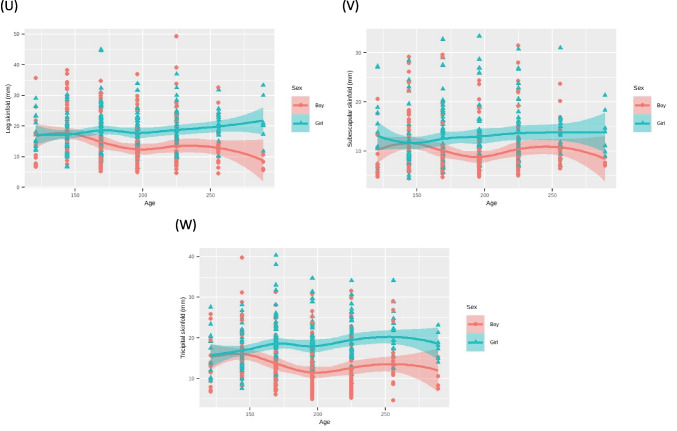
Anthropometric measures analyzed by age and sex. **A** Linear model considering height by age and sex. **B** Linear model considering weight by age and sex. **C** Linear model considering lean mass by age and sex. **D** Linear model considering fat mass by age and sex. **E** Linear model considering body water by age and sex. **F** Linear model considering humerus diameter by age and sex. **G** Linear model considering radio diameter by age and sex. **H** Linear model considering femur diameter by age and sex. **I** Linear model considering cephalic perimeter by age and sex. **J** Linear model considering contracted arm perimeter by age and sex. **K** Linear model considering arm perimeter by age and sex. **L** Linear model considering wrist perimeter by age and sex. **M** Linear model considering waist perimeter by age and sex. **N** Linear model considering hip perimeter by age and sex. **O** Linear model considering leg perimeter by age and sex. **P** Linear model considering pectoral skinfold by age and sex. **Q** Linear model considering bicipital skinfold by age and sex. **R** Linear model considering abdominal skinfold by age and sex. **S** Linear model considering suprailiac skinfold by age and sex. **T** Linear model considering thigh skinfold by age and sex. **U** Linear model considering leg skinfold by age and sex. **V** Linear model considering subescapular skinfold by age and sex. **W** Linear model considering tricipital skinfold by age and sex

In the bivariate analysis, linear regression models adjusted for age and sex were employed for each parameter in the study. Graphical representations illustrated changes in anthropometric measures with increasing age for each gender (Fig. 1, supplementary file).

To establish the predictive model, overweight (yes/no) was considered the outcome variable, according to the predefined criteria presented by Mueller et al., for each age/sex group and white population. A generalized additive model (GAM) logistic model was utilized to select predictors. Overweight served as the result and anthropometric measures, age, and sex acted as predictors. The optimal combination of variables was determined from the cross-validation technique, considering the area under the ROC curve (AUC) as the evaluation metric. This technique involves splitting the data set, such that the model is trained on several subsets of the data and evaluated on the remaining subset. This procedure is repeated several times, each time with a different combination of training and test sets. The AUC evaluation metric is calculated for each iteration. Finally, the results are averaged to obtain an overall evaluation of the model’s performance. This approach helps reduce the risk of overfitting and provides a more robust estimate of model performance on unseen data. In addition, it allows identifying the optimal combination of variables that maximizes the predictive capacity of the model, thus improving its generalization to new data sets. The R code is provided in Appendix 3.

The same methodology was applied to other routinely used pediatric care indices, such as BMI, adjusting for age and sex.

The diagnostic utility of the developed models was compared using the pROC software package [21]. For each model, the AUC with confidence intervals was calculated, along with sensitivity, specificity, positive and negative predictive values, true positive and true negative values, false positive and false negative values, accuracy, and positive and negative likelihood.

All analyses were conducted using R Studio statistical software package version 4.1.3 [22].

## Results

A total of 577 schoolchildren underwent measurement. Descriptive analysis results are presented in Table 1.

**Table 1:** Descriptive analysis

	**Mean (SD)**	**Number (%)**	**NA**
*N*	577	577	
Age	13.64		9
	(1.41)		
Weight (kg)	57.07		2
	(12.23)		
Height (cm)	165.90		2
	(60.53)		
Sex = Female (%)		281	
		(48.70)	
Nationality = Spanish (%)		545	
		(94.45)	
Fat mass (%)	20.71		7
	(8.38)		
Limit risk Female		31	
		(58.49)	
Limit risk Male		22	
		(41.51)	
Lean mass (%)	45.14		8
	(14.59)		
Body water (%)	33.26		9
	(8.21)		
Body mass index (kg/m^2^)	21.31		2
	(4.87)		
Classification (%)			2
Under weight		92	
		(18.8)	
Normal weight		334	
		(68.2)	
Overweight		53	
		(10.8)	
Obesity grade I		10	
		(2.0)	
Obesity grade II		1	
		(0.2)	
Obesity grade III		0	
		(0.0)	
Diameters (cm)			
Humerus	6.30		3
	(3.18)		
Radio	4.97		3
	(0.50)		
Femur	8.80		3
	(0.93)		
Perimeters (cm)			
Cephalic	55.77		2
	(4.81)		
Contracted arm	26.99		2
	(8.93)		
Arm	25.36		2
	(12.79)		
Wrist	16.26		3
	(16.81)		
Waist	69.20		2
	(9.64)		
Hip	88.46		2
	(9.57)		
Leg	39.29		14
	(108.39)		
Skinfolds (mm)			
Pectoral	8.41		2
	(4.35)		
Bicipital	9.51		2
	(5.35)		
Abdominal	17.53		1
	(8.62)		
Suprailiac	12.32		1
	(7.15)		
Thigh	22.03		1
	(10.58)		
Leg	16.06		1
	(7.62)		
Subscapular	11.49		1
	(5.81)		
Tricipital	15.78		1
	(6.73)		

^*^Data are mean(SD) or number (%)

During the data-cleaning and debugging process, 16 cases of influential data, 19 with values outside the usual ranges, and 52 with missing values were identified and subsequently excluded from the study, accounting for 15.08% of the initial sample. Consequently, the final analysis involved 490 schoolchildren.

The variables, including height, weight, lean mass, fat mass, water, humerus diameter, radius diameter, femur diameter, cephalic perimeter, contracted arm perimeter, arm perimeter, wrist perimeter, waist perimeter, hip perimeter, leg perimeter, pectoral skinfold, bicipital skinfold, abdominal skinfold, suprailiac skinfold, thigh skinfold, leg skinfold, subscapular skinfold, and tricipital skinfold, were plotted and analyzed with a breakdown by age and sex (Fig. 1).

The selection of predictor variables utilized an algorithm based on cross-validation techniques. This algorithm considered various combinations of independent variables and identified the model with the highest area under the curve (AUC). The selected variables included sex, weight, height, leg perimeter, and arm perimeter.

A second model was built with age, sex, and BMI due to its wide use in pediatric care. Two generalized additive regression models were constructed, and the coefficient estimates for these models are presented in Table 2.

**Table 2:** Coefficient estimates for generalized additive regression models

	**Model 1**^**a**^	**Model 2**^**b**^
Intercept	35.75*	− 17.72**
	(14.58)	(2.81)
Sex	1.31*	1.07*
	(0.53)	(0.45)
Age		− 0.06
		(0.15)
Weight	4.66**	
	(0.83)	
Height	− 4.80**	
	(1.09)	
Leg perimeter	− 4.29*	
	(1.73)	
Arm perimeter	2.19	
	(1.54)	
Body mas index		0.66**
		(0.09)
Num. obs.	490	490
Nagelkerke *R*^2^ adjust.	0.49	0.46
Generalized AIC	223.99	248.32

*p < 0.05;  **p < 0.001

^a^Model 1: outcome = Fat mass (%), independent variables = sex + weight + height + leg perimeter + arm perimeter

^b^Model 2: outcome = Fat mass (%), independent variables = sex + body mass index

Sex was incorporated as a predictor in all two models, and significance was observed in both models. Table 3 provides the exponential transformation of the coefficient estimates associated with the qualitative variable, sex. This transformation yields the odds ratios (ORs) along with their corresponding confidence intervals (CIs).

**Table 3:** Odds ratios (ORs) and confidence intervals (CIs) for sex in generalized additive regression models

	**Model 1**^**a**^	**Model 2**^**b**^
	OR (SD)	OR (SD)
Sex	3.70 (0.53)*	2.92 (0.34)*

^*^p < 0.05

^a^Model 1: outcome = Fat mass (%), independent variables = sex + weight + height + leg perimeter + arm perimeter

^b^Model 2: outcome = Fat mass (%), independent variables = sex + body mass index

To assess and compare the models, ROC curves were constructed, and the area under the curve (AUC) along with their confidence intervals (CIs) was calculated. The AUC values for each model are detailed in Table 4: model 1 (0.957, 95% CI: 0.928 to 0.986) and model 2 (0.944, 95% CI: 0.903 to 0.983).

**Table 4:** Diagnostic utility for each model

	**Model 1**	**Model 2**
Sensitivity	0.93 (0.83–1.00)	0.93 (0.81–1.00)
Specificity	0.91 (0.83–0.96)	0.90 (0.80–0.97)
Positive predictive values	0.48 (0.34–0.68)	0.46 (0.31–0.74)
Negative predictive values	0.99 (0.98–1.00)	0.99 (0.98–1.00)
True positive rate^a^	92.86 (85.71; 100)	92.85 (84.00–100)
True negative rate^a^	90.40 (85.14; 95.40)	89.96 (83.17–96.51)
False positive rate^a^	9.60 (4.60; 14.85)	10.04 (0.03–16.83)
False negative rate^a^	7.14 (0.00; 14.29)	7.14 (0.00–16.00)
Accuracy	0.91 (0.84–0.96)	0.90 (0.82–0.96)
Positive likelihood ratio	9.90 (4.85–23.58)	9.24 (4.17–34.46)
Negative likelihood ratio	0.07 (0.00–0.20)	0.08 (0.00–0.24)
AUC	0.957 (0.928–0.986)	0.944 (0.903–0.984)

Model 1: outcome = Fat mass (%), independent variables = sex + weight + height + leg perimeter + arm perimeter. Model 2: outcome = Fat mass (%), independent variables = sex +  body mass index. Data are estimates (confidence interval)

^a^Data expressed as percentages

**Fig. 2:**
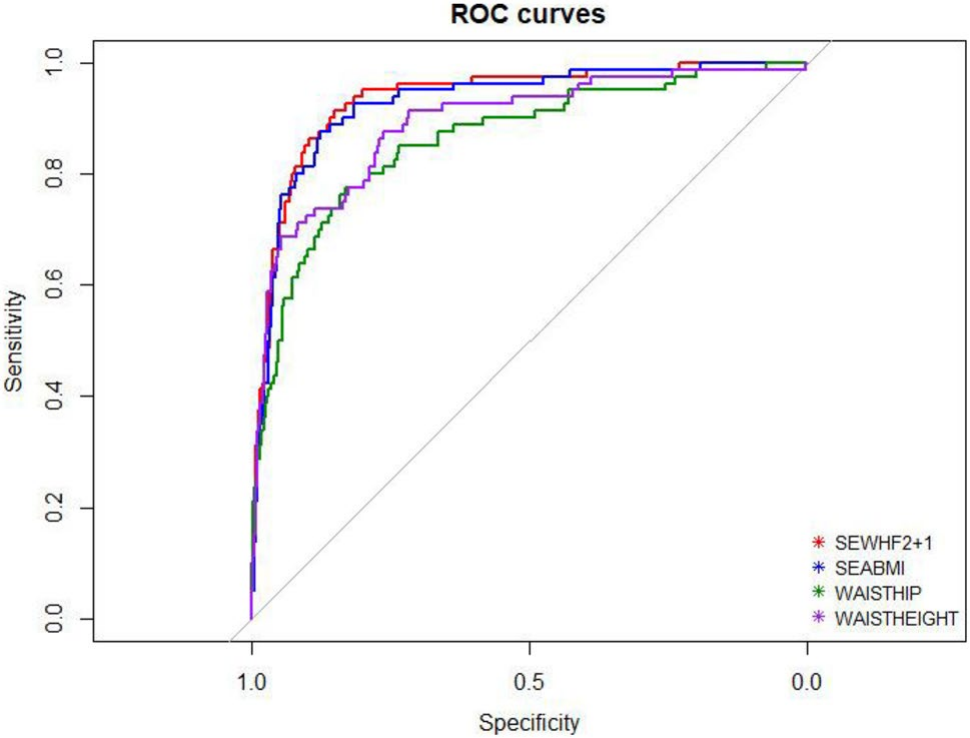
ROC curves associated with the models

Figure 2 provides visual representations of the ROC curves for each model.

According to the roc.test function of the pROC package, there were no significant differences between the ROC curves associated with the model 1 and model 2, respectively, with a CI of 0.05.

Sensitivity, specificity, positive and negative predictive values, true positive and true negative values, false positive and false negative values, accuracy, and positive and negative likelihood ratios are summarized in Table 4.

## Discussion

The objective of this study was to develop a mathematical formula based on anthropometric measures for estimating fat mass composition in the child population, aiming to improve the classification of obesity risk in primary care pediatric clinics. Additionally, an online calculator was designed to facilitate its use https://hced.isaudegal.es/shiny/overweight/.

The developed model 1 utilized a GAM multivariate regression and includes five variables: sex, weight, height, leg perimeter, and arm perimeter. This model was compared with the other model, Model 2 includes sex, age, and BMI. These models yielded *R*^2^ values of 0.49 and 0.46, respectively.

Models with an area under the curve (AUC) exceeding 0.75 are deemed suitable for clinical practice [23]. AUC values for models 1 and 2 were 0.957 (0.928–0.986) and 0.944 (0.903–0.984), respectively, indicating high discriminatory power for diagnostic use.

Several equations for calculating total body density and fat mass rely on aggregations of cutaneous skinfold thickness [24–31], typically involving four skinfolds (biceps + triceps + subscapular + suprailiac), unlike the models proposed in this study. Goran et al. proposed a model in 1996 to estimate DXA-measured fat mass, incorporating the thickness of two cutaneous skinfolds—subscapular and triceps—along with body weight, sex, and height/resistance. This model yielded an *R*^2^ of 0.91 and a SEE of 0.94 kg of fat mass in direct regression analysis [15]. It is noteworthy that none of these models incorporate measures of the lower limbs, despite their importance in terms of functionality in pediatric ages [31]. Additionally, these models were based on small-sized samples at pediatric age [25].

Wong et al. [32] evaluated the concordance between eight commonly used cutaneous skinfold equations, using a multicompartmental model to predict the percentage of body fat in 72 White and 40 Afro-American girls aged 11 through 15 years. In the Bland–Altman analysis, quadratic equations showed closer agreement with the fat mass measure of the four-compartment model. Slaughter’s equation was identified as the one that best estimated fat mass, and unlike the others, it considered leg skinfold. Similar relative biases and 95% concordance limits were obtained when thickness measures of cutaneous triceps and calf skinfolds were used instead of those of cutaneous subscapular and bicep skinfolds in Slaughter et al.’s equation [32].

There is limited availability of sensitivity and specificity data on different equations published for anthropometric measures. In 2014, Wohlfahrt-Veje et al. [33] analyzed the concordance between various anthropometric measures and body fat values estimated by DXA in 2647 Danish children. The highest correlation was observed with cutaneous skinfolds in identifying children with excess fat (*R* 0.86), compared to estimates by waist-hip index and BMI (*R* 0.78 and 0.69, respectively). Sensitivity and specificity values were 79.5 and 93.8 for the concordance of cutaneous skinfolds, 75.9 and 90.3 for BMI, and 59.2 and 95.4 for waist-hip index. Generally, our models displayed higher sensitivity and similar specificity compared to Wohlfahrt-Veje’s findings. Model 1 exhibited a sensitivity and specificity of 0.93 and 0.91, respectively, versus 79.5 and 93.8 for the cutaneous skinfold model. Model 2 yielded values of 93 and 90 versus 75.9 and 90.3 for BMI.

The gold standard for comparing measures of body composition relies on multicompartmental models encompassing estimates of weight, body volume, body density, bone mineral content, and total body water. However, implementing such models is not practically feasible in clinical or community settings. An alternative method, of particular significance for assessing body fat in both individual cases and epidemiological groups, is bioelectrical impedance analysis. This method stands out as the most widely employed, given its cost-effectiveness, user-friendly nature, and non-invasive approach to determining fat or lean mass. Additionally, it is recognized as a valid tool for longitudinal follow-up [34]. The pediatric population has demonstrated excellent concordance between electrical bioimpedance and DXA, making it a valuable tool for validating anthropometric data [35, 36].

Limitations of the study include the elapsed time between data collection and analysis. However, this is unlikely to significantly affect the relationship between parameters. Replicating the analysis in larger, international populations for external validation is recommended. The use of GAM models, while offering accurate predictions, requires calculators for interpretation.

Strengths of the study include the use of a representative sample of the child population aged 11 to 17 years, allowing extrapolation of results to similar populations. The analysis was conducted without imputation, as missing values were minimal and unrelated to the outcome variable. The study also employed an in-house algorithm to optimize model selection for the highest AUC. The cross-validation technique with *k* iterations not only contributes to the internal reliability of the study, but also represents a crucial strength in providing external validity. This methodological approach gives the model significant robustness by subjecting it to multiple training and evaluation cycles with different data sets, allowing a more precise evaluation of the model’s performance and increasing its reliability and applicability.

Furthermore, another strong point is that the calculations and values obtained are based on the percentage of body fat rather than on BMI, thus eliminating any bias that this estimation may have.

Model 1 and model 2 show a slight difference between the two with model 2 requiring fewer anthropometric measures. Although, it is noteworthy that both models are adjusted by sex and age, saving time and avoiding errors associated with the use of percentiles of the growth curve. An online calculator incorporating these models was created, streamlining calculations and classification of children concerning overweight/obesity in clinical practice.

In conclusion, the study proposes practical models for estimating the risk of overweight and obesity in children, with the BMI model recommended for routine clinical practice in primary care pediatric clinics due to its simplicity and efficiency. The developed online calculator automates calculations, optimizing time and enhancing reliability in clinical practice. Both predictive models show very high parameters of diagnostic utility.

## Conclusions

Two predictive models, with the 85th percentile of fat mass as the gold standard, built with basic anthropometric measures, show very high diagnostic utility parameters. Their calculation is facilitated by a complementary online calculator.

The development and presentation of the calculator stand out as a significant contribution of this study, potentially offering a substantial impact on the efficiency of primary care pediatric clinics. This is particularly relevant in environments lacking impedance meters, as the instruments for skinfolds and perimeters are relatively inexpensive. The practical models proposed, especially the BMI model, provide a valuable tool for routine clinical practice, offering simplicity, efficiency, and accessibility in the assessment of overweight and obesity risk in children. The online calculator, derived from these models, has the potential to enhance the speed and reliability of such assessments, thereby facilitating timely interventions and contributing to the overall effectiveness of pediatric care.

### Supplementary Information

Below is the link to the electronic supplementary material.

Supplementary file1 (DOCX 17 KB)

Supplementary file2 (DOCX 14 KB)

Supplementary file3 (DOCX 17 KB)

## Data Availability

The datasets used and/or analysed during the current study are available from the corresponding author on reasonable request.

## Declarations

### Disclaimer

The funderswere not involved in the study design, collection, analysis, and interpretation of data, the writing of this article or the decision to submit it for publication.
