# Supplementary material for: BODYFAT: a new calculator to determine the risk of being overweight validated in Spanish children between 11 and 17 years of age
Source: Eur J Pediatr. 2024 Jun 19;183(9):3885–95. doi: 10.1007/s00431-024-05596-2 (PMC11322224; doi:10.1007/s00431-024-05596-2)
Supplement: Supplementary file 1 — Supplementary file1 (DOCX 17 KB) [file 431_2024_5596_MOESM1_ESM.docx]

**APPENDIX 1**

**Measurement protocols**

1. Height: Defined as the distance in centimetres from the vertex (the highest point of the head) to the ground. The subject shall stand with feet together, arms along the body, buttocks and against the scale and head on the Frankfort plane (when paralel to the ground, the imaginary line extending from the most inferior point of the orbital margin to the highest point of the external auditory canal (tragion)).
2. Weight: The subject shall stand on the centre of the weighing platform of the scale, evenly distributing his/her weight on both feet and unsupported. The subject must be weighed in his/her underwear. The subject should be weighed at the same time of the day. The best time of the day is early morning before breakfast.
3. Folds: To obtain body composition values as reliable and valid as possible, the following protocol shall be followed:

Carefully locate the anatomical landmarks, following the indications for each skinfold. Once located, the exact measuring point shall be marked with a skin marker so as to always measure from the same point.

The fold is grasped between the index finger and thumb of the left hand, lifting a double layer of skin and the underlying fatty tissue and pulling slightly away from the body until the measurement is done. The caliper shall be applied with the right hand 1-2 cm proximal to the point where the fold is taken and perpendicular to the fold.

Each fold shall be determined in triplicate. The value shall be the average of the three measurements. If some measurement diverges considerably from the average (i.e., 2 standard deviations), the measurement shall be repeated. Measurements shall be repeated after completing the first measurement of all the folds. Otherwise, in measurements repeated at a very short interval, lower readings shall be obtained due to the compression of the skin caused by the previous measurement.

Measurements must be taken always by the same observer. If measures are taken by more than one observer, the inter-observer level of reliability must be established.

Readings of skinfold thickness must be carried out 4 seconds after the skinfold

caliper has been released, thus partly preventing the variability associated with

differences in skin compressibility.

**Location of measurement points**

Skinfolds:

1. Tricipital: Measured in the posterior aspect of the arm, midway between the

acromion and the upper end of the radial head. It must be taken vertically.

1. Subescapular: Measured 1-2 cm proximal from the inferior angle of the

scapula. The direction of the skinfold must be oblique, downwards and away, at

about 45 degrees to the horizontal.

1. Suprailiac: It is located in the intersection of a pseudo-vertical

line running through the anterior superior iliac spine and the anterior edge of the

axilla on the same side and a horizontal line from the uppermost edge of the iliac

crest. The fold is oblique, downwards and inwards, at about 45 degrees to the

horizontal.

1. Abdominal: Taken at the level of the umbilical scar, about 3-5 cm to the right.

Its direction is vertical and runs parallel to the longitudinal axis of the body.

1. Pectoral: Measured over the line extending from the axilla to the nipple, as close

as possible to the axillary fold.

1. Thigh: Located midway along the line drawn between the inguinal

fold and the upper part of the kneecap. It is measured vertically, and the subject

shall be either sitting or standing, but the leg shall be raised on a stool so that the

knee will be flexed at 90 degrees.

1. Leg: Taken at the level of the maximum circumference of the leg on

its medial surface. It is vertical and runs parallel to the longitudinal axis of the leg.

It is measured while the subject stands with the knee flexed at 90 degrees, resting

his/her foot on a stool.

Diameters:  Both arms of the anthropometer or pachymeter are held between index finger and thumb. The bony landmark is located with the middle finger. Firm pressure must be applied to compress soft tissues.

1. Radio: The distance between the styloid process of radius and ulna. The anthropometrist shall face the subject, who shall be seated with his/her forearm in pronation over the thigh and the hand bent at the wrist to an angle of about 90 degrees. The arms of the pachymeter point downwards in the bisector of the wrist angle.
2. Humerus: The distance between the lateral and medial epicondyle of the humerus. The subject shall place his/her arm in an anterior and horizontal position, with elbow bent at a right angle and forearm in supination. The arms of the pachymeter point downwards at an angle of 45 degrees to the horizontal.
3. Femur: The distance between the lateral and medial condyle of the femur. The subject is seated with knees bent at a 90-degree angle. The arms of the pachymeter point downwards at an angle of 45 degrees to the horizontal.

Perimeters: The anthropometrist shall hold the left end of the tape with his/her left hand and the opposite end or the tape reel with his/her right hand. The tape is placed on the area at the required level, without compression of the soft tissues and at right angles to the longitudinal axis of the body segment being measured.

1. Contracted arm: It is measured at the area of the maximum circumference of the arm. The subject shall lift his/her arm at right angles to the horizontal, in antepulsion and with the elbow bent to about 45 degrees. He/she shall then carry out a maximum contraction of the flexor muscles of the arm. Girth is measured on the right arm.
2. Arm: This perimeter is measured midway along the acromion-radial distance.
3. Hip: The perimeter at the level of the largest gluteal circumference, approximately above the pubic symphysis.
4. Waist: It can also be called Abdominal 1. It is located at the point where the circumference of the abdomen is smaller, approximately midway between the costal margin and the iliac crest.
5. Cephalic: Maximum perimeter of the head when the tape is placed above the glabella (midpoint between the eyebrows).
6. Wrist: Distal girth of the wrist, which coincides with the minimum girth of the forearm. The subject flexes his/her elbow, with the palm of his/her hand facing upward.
7. Leg: Measured at the area of maximum circumference of the leg. The subject stands with feet slightly apart and weight evenly distributed between both feet. Measurement is taken on the lateral side of the leg.
8. Impedance measurement: Results are automatically shown by the measurement machine after a few seconds of contact with the electrodes.
