# Supplementary material for: BODYFAT: a new calculator to determine the risk of being overweight validated in Spanish children between 11 and 17 years of age
Source: Eur J Pediatr. 2024 Jun 19;183(9):3885–95. doi: 10.1007/s00431-024-05596-2 (PMC11322224; doi:10.1007/s00431-024-05596-2)
Supplement: Supplementary file 2 — Supplementary file2 (DOCX 14 KB) [file 431_2024_5596_MOESM2_ESM.docx]

**APPENDIX 2**

**Measuring instruments**

Measuring instruments used, duly homologated as per the recommendations of the International Society for the Advancement of Kinanthropometry (ISAK), were as follows:

1. SECA 877 SCALE, to measure body mass (weight), accurate to 100 g.
2. MEASURING ROD Leicester Height Measure: height measuring instrument accurate to 1 mm.
3. MEASURING TAPE by Kawe, flexible and metallic to prevent mistakes by stretching and accurate to 1 mm.
4. TANITA BODY BIOIMPEDANCE ANALYZER MODEL BC-418 MA. Based on the principle of impedance (Z), which analyses the response of a fluid-filled cylinder to the passage of an electrical current.
5. Harpenden PLICOMETER (Skinfold Caliper RH15 9LR, England). It is a caliper used to assess the skinfold thickness. It exerts a constant pressure no matter the aperture and is accurate to less than 0,2 mm.
6. Holtain PACHYMETER. Instrument for measuring bone diameters accurate to 1 mm.
7. SKIN MARKER: Used to mark the exact measuring point. It must be suitable for marking skin and hypoallergenic.
8. IMPEDANCE METER: Based on the principle of impedance (Z), which analyses the response of a fluid-filled cylinder to the passage of an electrical current.
