# Supplementary material for: BODYFAT: a new calculator to determine the risk of being overweight validated in Spanish children between 11 and 17 years of age
Source: Eur J Pediatr. 2024 Jun 19;183(9):3885–95. doi: 10.1007/s00431-024-05596-2 (PMC11322224; doi:10.1007/s00431-024-05596-2)
Supplement: Supplementary file 3 — Supplementary file3 (DOCX 17 KB) [file 431_2024_5596_MOESM3_ESM.docx]

**APPENDIX 3**

**R code of the algorithm**

***#----Gam model construction***

*modelo=gam(riesgom~Sexo+Edad+Peso+Altura+DiamHU+DiamRA+DiamFE+PeriCE+PeriBC+PeriBR+PeriMUÑ+PeriCI+PeriCA+PeriPI+meanPE+meanBI+meanAB+meanSP+meanMU+meanPI+meanBS+meanTP,family="binomial",data=datos)*

*summary(modelo)*

***#----Associated ROC curve***

*A_gam=roc(datos$riesgom,predict(modelo,type="response"))*

*plot(A_gam,print.auc=TRUE,main="GAM model")*

*Y="riesgom"; nombres=setdiff(names(datos),Y)*

*ret=c("threshold", "specificity", "sensitivity",*

*"accuracy", "fn", "fp", "npv", "ppv" )*

*res_gam=coords(A_gam,"best",best.method="closest.topleft",ret=ret)*

*AUC_gam=function(X,Y,datos,K=2){*

*set.seed(89)*

*formula=paste(Y,"~")*

*for (j in 1:length(X)) {*

*aux=datos[,X[j]]*

*if (is.factor(aux)) {formula=paste(formula,"+",X[j],sep="")}*

*else {formula=paste(formula,"+",X[j],sep="")}}*

*formula=as.formula(formula)*

*n=nrow(datos)*

*auc=numeric(K)*

*for (k in 1:K){*

*ii=sample(n,size=0.70*n)*

*training=datos[ii,]; test=datos[-ii,]*

*modelo=gam(formula,family="binomial",data=training)*

*pred=predict(modelo,type="response",newdata=test)*

*A=roc(test[,Y],pred); auc[k]=A$auc}*

*mean(auc)}*

***#------ Combinations***

*library(pROC)*

*library(caTools)*

*AUC=NULL; XX=NULL*

*for (p in 1:5) {*

*X=combs(nombres, p)*

*for (i in 1:nrow(X)) XX=rbind(XX,is.element(nombres,X[i,]))*

*AUC=c(AUC,apply(X,1,AUC_gam,Y=Y,datos=datos))}*

*colnames(XX)=nombres;*

*p=apply(XX,1,sum)*

*res=data.frame(nvar=p,XX,AUC=AUC)*

*res_ordenado_por_auc <- res[order(res$AUC,decreasing = TRUE), ]*

*View(res_ordenado_por_auc)*

*res_gam=res_ordenado_por_auc*

*View(res_gam[1:25,])*

*summary(res_gam[1])*
